# Supplementary material for: Stability of β-lactam antibiotics in bacterial growth media
Source: PLoS One. 2020 Jul 20;15(7):e0236198. doi: 10.1371/journal.pone.0236198 (PMC7371157; doi:10.1371/journal.pone.0236198)
Supplement: S1 Fig — Plate reader growth curves for E. coli MG1655 in both MOPSgluMIN and MOPSgluRDM. OD refers to the optical density at 600 nm. The error bars represent the standard error of the mean between the 11 replicates for each condition. (PDF) [file pone.0236198.s001.pdf]

S1 Fig.

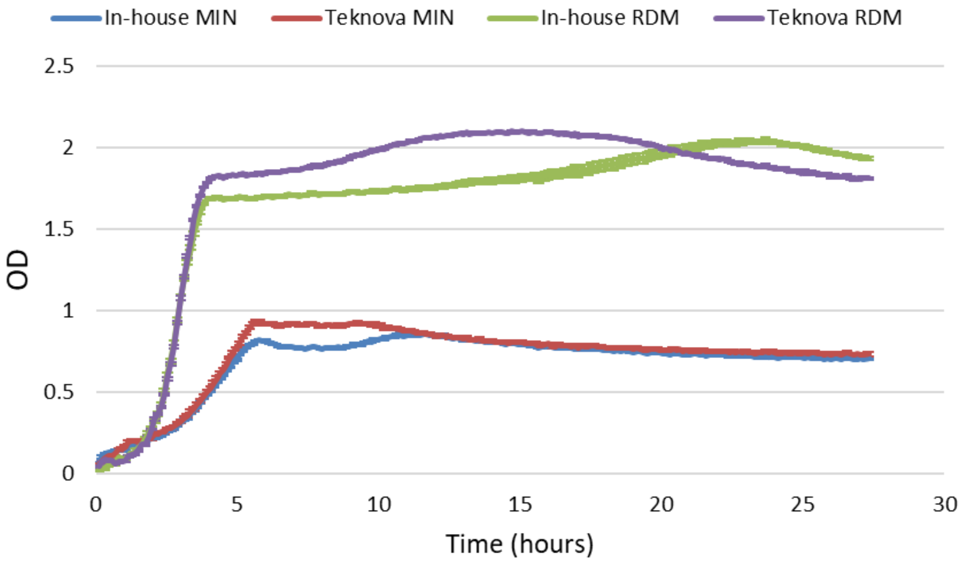

Comparison of growth dynamics for in-house and Teknova MOPS media. Plate reader growth curves for *E. coli* MG1655 in both MOPSgluMIN and MOPSgluRDM. OD refers to the optical density at 600 nm. The error bars represent the standard error of the mean between the 11 replicates for each condition.
