# Supplementary material for: Stability of β-lactam antibiotics in bacterial growth media
Source: PLoS One. 2020 Jul 20;15(7):e0236198. doi: 10.1371/journal.pone.0236198 (PMC7371157; doi:10.1371/journal.pone.0236198)
Supplement: S2 Fig — Diagram of the light path used to obtain the Raman spectra from a top-down perspective. The âŠ— and ⊙ symbols represent the electric wave vector going into, and coming out of, the page, respectively. The sample is held in a cuvette within a copper block, with four holes, through which the scattered laser light can be collect (and amplified) perpendicular to the incident light. The copper block is also used to achieve temperature control of the sample. (PDF) [file pone.0236198.s002.pdf]

S2 Fig.

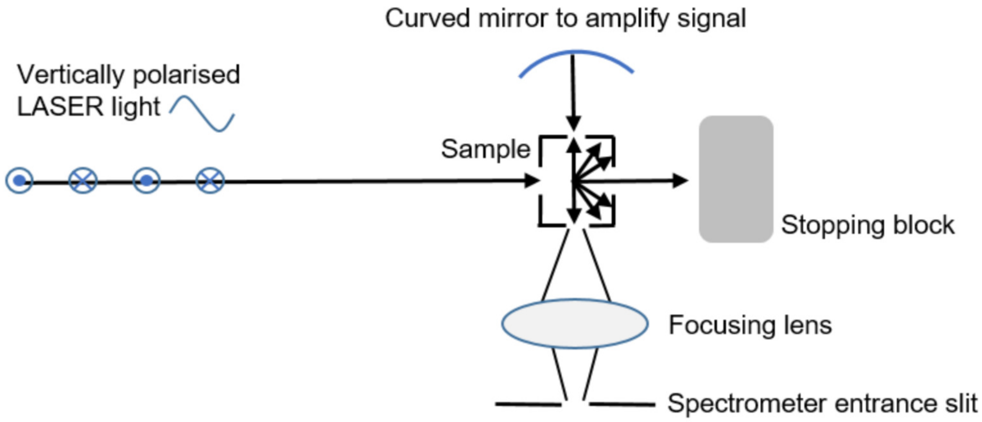

Raman spectroscopy set-up.
Diagram of the light path used to obtain the Raman spectra from a top-down perspective. The $\otimes$ and $\odot$ symbols represent the electric wave vector going into, and coming out of, the page, respectively. The sample is held in a cuvette within a copper block, with four holes, through which the scattered laser light can be collect (and amplified) perpendicular to the incident light. The copper block is also used to achieve temperature control of the sample.
