# Supplementary material for: Stability of β-lactam antibiotics in bacterial growth media
Source: PLoS One. 2020 Jul 20;15(7):e0236198. doi: 10.1371/journal.pone.0236198 (PMC7371157; doi:10.1371/journal.pone.0236198)
Supplement: S3 Fig — A, B: Identical population dynamics are observed for E. coli MG1655 (solid lines) and RJA002 (dashed lines). A: Population dynamics of MG1655 and RJA002 in LB with mecillinam. B: Population dynamics of MG1655 and RJA002 in MOPSgluRDM with cefotaxime. C: Regrowth is observed for E. coli RJA002 on LB growth medium at around 1000 minutes for mecillinam concentrations of 0.375μg/ml up to 6μg/ml. D: Regrowth is observed for E. coli MG1655 on MOPSgluCAA at around 400 minutes for mecillinam concentrations of 0.094μg/ml-0.375μg/ml. E: Regrowth is observed for E. coli MG1655 on MOPSglycRDM (glyc signifies 20% w/v glycerol is used instead of glucose) after 600 minutes for mecillinam concentrations of 0.094μg/ml-0.75μg/ml, F: Regrowth is observed for E. coli MG1655 on MOPSglycCAA after 800 minutes for mecillinam concentrations of 0.094μg/ml-0.375μg/ml. In every panel the mecillinam concentrations displayed are listed in the legend with units of μg/ml, and the shading represents the standard deviation between the averaged 2-4 replicates. (PDF) [file pone.0236198.s003.pdf]

S3 Fig.

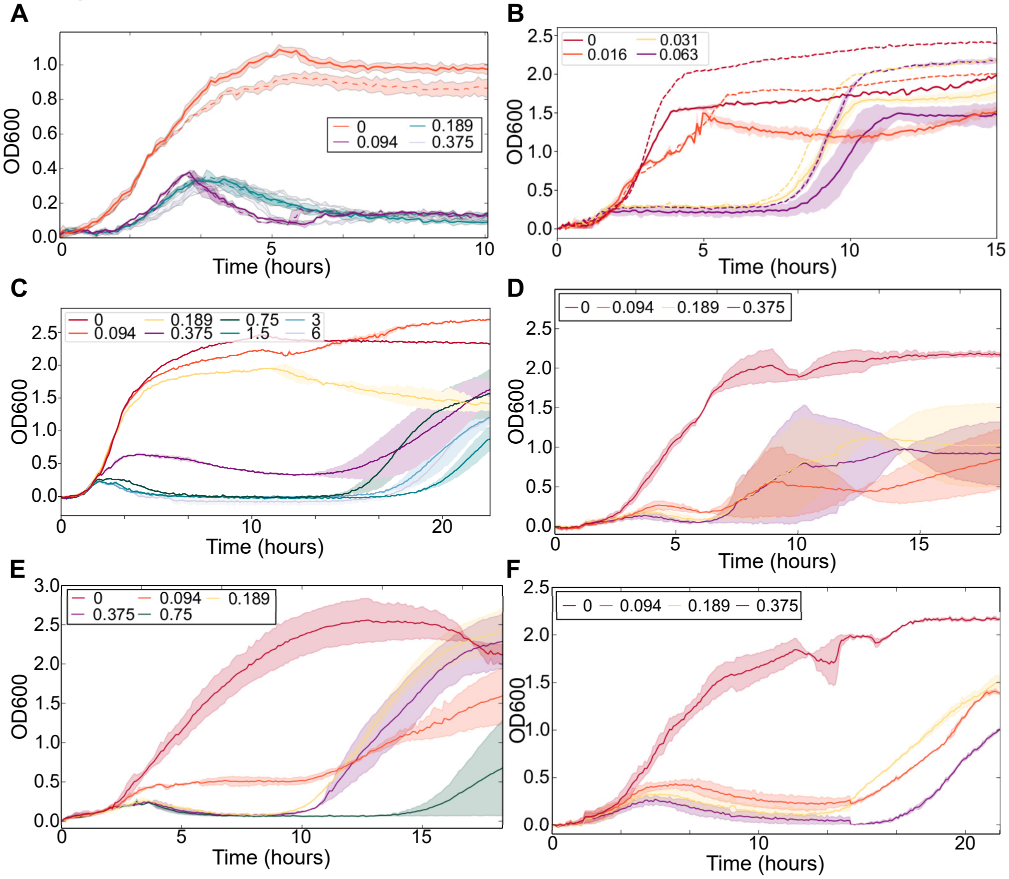

Growth curves showing regrowth for *E. coli* MG1655 and RJA002 in the presence of mecillinam or cefotaxime on various media.

A, B: Identical population dynamics are observed for *E. coli* MG1655 (solid lines) and RJA002 (dashed lines). A: Population dynamics of MG1655 and RJA002 in LB with mecillinam. B: Population dynamics of MG1655 and RJA002 in MOPSgluRDM with cefotaxime. C: Regrowth is observed for *E. coli* RJA002 on LB growth medium at around 1000 minutes for mecillinam concentrations of 0.375μg/ml up to 6μg/ml. D: Regrowth is observed for *E. coli* MG1655 on MOPSgluCAA at around 400 minutes for mecillinam concentrations of 0.094μg/ml-0.375μg/ml. E: Regrowth is observed for *E. coli* MG1655 on MOPSglycRDM (glyc signifies 20% w/v glycerol is used instead of glucose) after 600 minutes for mecillinam concentrations of 0.094μg/ml-0.75μg/ml, F: Regrowth is observed for *E. coli* MG1655 on MOPSglycCAA after 800 minutes for mecillinam concentrations of 0.094μg/ml-0.375μg/ml. In every panel the mecillinam concentrations displayed are listed in the legend with units of μg/ml, and the shading represents the standard deviation between the averaged 2-4 replicates.
