# Supplementary material for: Stability of β-lactam antibiotics in bacterial growth media
Source: PLoS One. 2020 Jul 20;15(7):e0236198. doi: 10.1371/journal.pone.0236198 (PMC7371157; doi:10.1371/journal.pone.0236198)
Supplement: S4 Fig — A, B, C: Plots of time to reach OD threshold of 0.75. The red curve corresponds to the black curve (immediate inoculation) shifted by 1 log2 unit. The blue and green curves are for inoculations delayed by 2, 3, 4 or 6 hours. Shading around the lines is representative of the standard deviation between the two averaged replicates. A: MOPSgluRDM at 37° and pH 6.5. As the green curve (inoculation delayed by 4 hours) coincides with the red curve the half-life for mecillinam can be approximated at around 4 hours. B: MOPSgluRDM at 34°C and pH 7.4. As the blue curve coincides with the red curve the half-life for mecillinam is around 2 hours. C: MOPSgluRDM at 34° and pH 6.5. Neither the blue nor the green curve reaches the red curve, therefore the half life is greater than 6 hours. D, E: Raman spectroscopy confirms decreased degradation of mecillinam in MOPSgluRDM at 34°, pH 6.5. C: Raman spectra for the region between 1000 and 1400 wavenumbers, recorded at times 0 to 47 hours (time in hours indicated by colour as shown in the legend). Successive curves are shifted upwards for clarity. The peak of interest is highlighted by the vertical black line. E: The relative peak height at 1278 cm−1 as a function of time is fit to an exponential decay, which gives a half life of 7.8±3.1 hours. The peak height at 1278 cm−1 was measured relative to a stable portion of the Raman spectrum (1261 − 1266 cm−1)to remove any variations due to changing background signals between timepoints. (PDF) [file pone.0236198.s004.pdf]

S4 Fig.

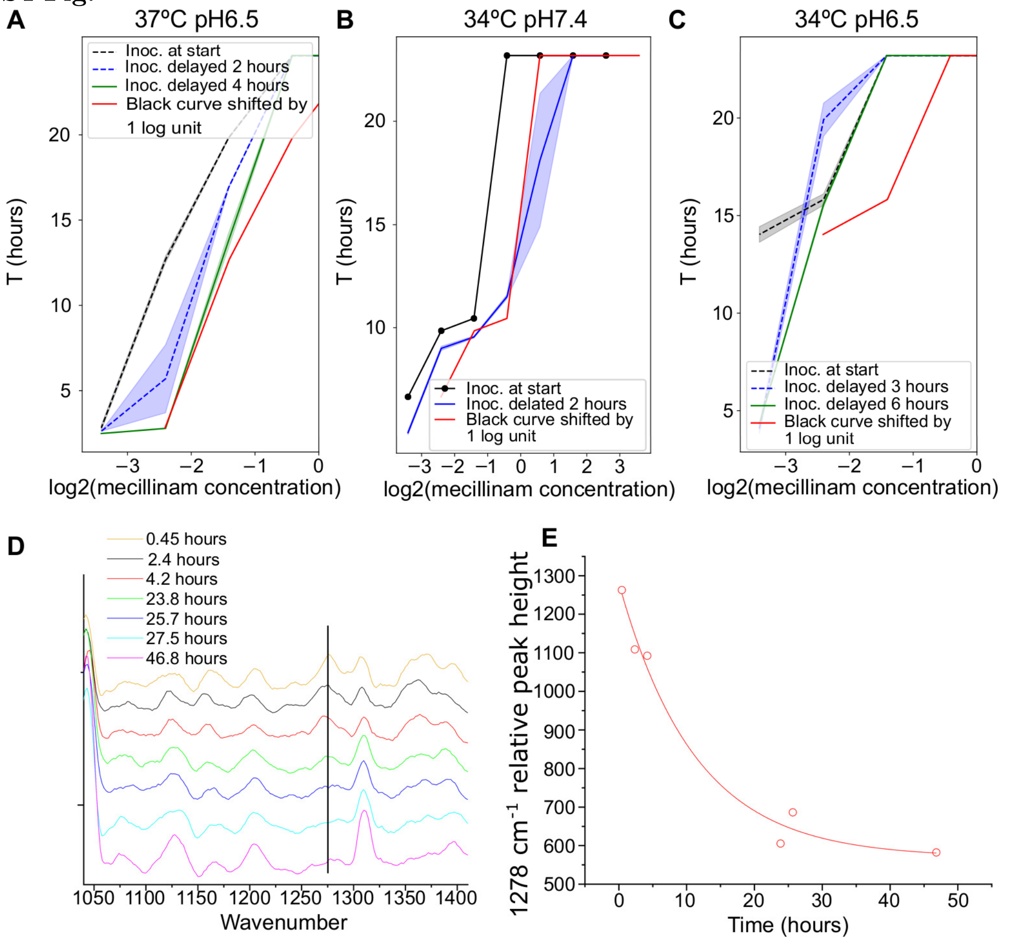

Delay time assay and Raman spectra for mecillinam in MOPSgluRDM at shifted pH and temperature

A, B, C: Plots of time to reach OD threshold of 0.75. The red curve corresponds to the black curve (immediate inoculation) shifted by 1 $\log_2$ unit. The blue and green curves are for inoculations delayed by 2, 3, 4 or 6 hours. Shading around the lines is representative of the standard deviation between the two averaged replicates. A: MOPSgluRDM at 37° and pH 6.5. As the green curve (inoculation delayed by 4 hours) coincides with the red curve the half-life for mecillinam can be approximated at around 4 hours. B: MOPSgluRDM at 34°C and pH 7.4. As the blue curve coincides with the red curve the half-life for mecillinam is around 2 hours. C: MOPSgluRDM at 34° and pH 6.5. Neither the blue nor the green curve reaches the red curve, therefore the half life is greater than 6 hours. D, E: Raman spectroscopy confirms decreased degradation of mecillinam in MOPSgluRDM at 34°, pH 6.5. C: Raman spectra for the region between 1000 and 1400 wavenumbers, recorded at times 0 to 47 hours (time in hours indicated by colour as shown in the legend). Successive curves are shifted upwards for clarity. The peak of interest is highlighted by the vertical black line. E: The relative peak height at 1278 cm^{-1} as a function of time is fit to an exponential decay, which gives a half life of 7.8 ± 3.1 hours.The peak height at 1278 cm^{-1} was measured relative to a stable portion of the Raman spectrum (1261 - 1266 cm^{-1})to remove any variations due to changing background signals between timepoints.
