# Supplementary material for: Stability of β-lactam antibiotics in bacterial growth media
Source: PLoS One. 2020 Jul 20;15(7):e0236198. doi: 10.1371/journal.pone.0236198 (PMC7371157; doi:10.1371/journal.pone.0236198)
Supplement: S5 Fig — A: Bioassay measurements: growth curves for E. coli strain RJA002 in LB at pH 7.0, 37°C, for cultures inoculated at time zero (solid lines) and after a delay period of 2 hours (dashed lines). The different colours correspond to different (initial) concentrations of mecillinam, as displayed in the legend, in μg/ml. B: Bioassay analysis—the time T at which the growth curves reach an OD of 0.75 is plotted as a function of the initial antibiotic concentration. The black data correspond to inoculation at time zero; the blue data to inoculation after a 2 hour delay; the green data to inoculation after a 4 hour delay; and the turquoise data to inoculation after a 6 hour delay. The red line shows the black data, shifted by one unit on the log2 scale; since the green and turquoise data fall onto the red line, the degradation half-life is between 4 and 6 hours. In both panels A and B, the shaded areas represent the standard deviation between two replicates. (PDF) [file pone.0236198.s005.pdf]

S5 Fig.

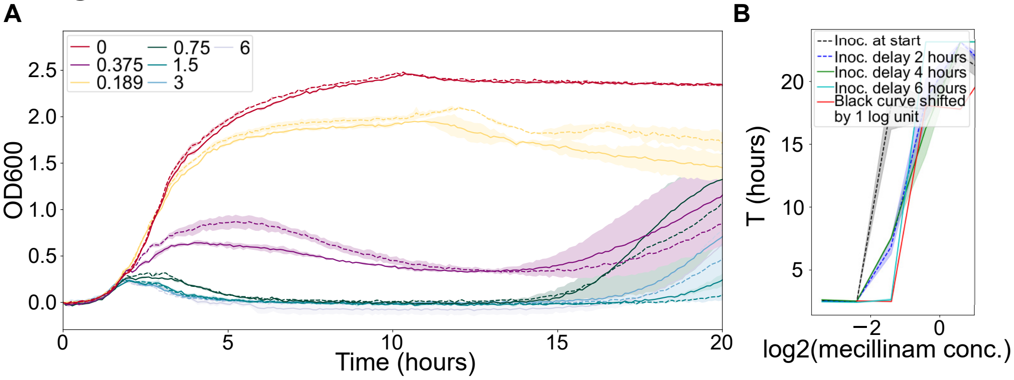

Delay time bioassay results for mecillinam in LB at 37° C and pH 7.
A: Bioassay measurements: growth curves for *E. coli* strain RJA002 in LB at pH 7.0, 37° C, for cultures inoculated at time zero (solid lines) and after a delay period of 2 hours (dashed lines). The different colours correspond to different (initial) concentrations of mecillinam, as displayed in the legend, in μg/ml. B: Bioassay analysis - the time T at which the growth curves reach an OD of 0.75 is plotted as a function of the initial antibiotic concentration. The black data correspond to inoculation at time zero; the blue data to inoculation after a 2 hour delay; the green data to inoculation after a 4 hour delay; and the turquoise data to inoculation after a 6 hour delay. The red line shows the black data, shifted by one unit on the $\log_2$ scale; since the green and turquoise data fall onto the red line, the degradation half-life is between 4 and 6 hours. In both panels A and B, the shaded areas represent the standard deviation between two replicates.
