# Supplementary material for: Stability of β-lactam antibiotics in bacterial growth media
Source: PLoS One. 2020 Jul 20;15(7):e0236198. doi: 10.1371/journal.pone.0236198 (PMC7371157; doi:10.1371/journal.pone.0236198)
Supplement: S6 Fig — Regrowth is only observed in a narrow cefotaxime concentration range between 0.031 and 0.125μg/ml cefotaxime. Each curve is the average of 2-4 replicates and the shaded areas represent the standard deviation. The blue curve (0.25 μg/ml Ctx.) shows complete inhibition of growth over time, while the orange curve (0.016 μg/ml Ctx.) shows no inhibition. (PDF) [file pone.0236198.s006.pdf]

S6 Fig.

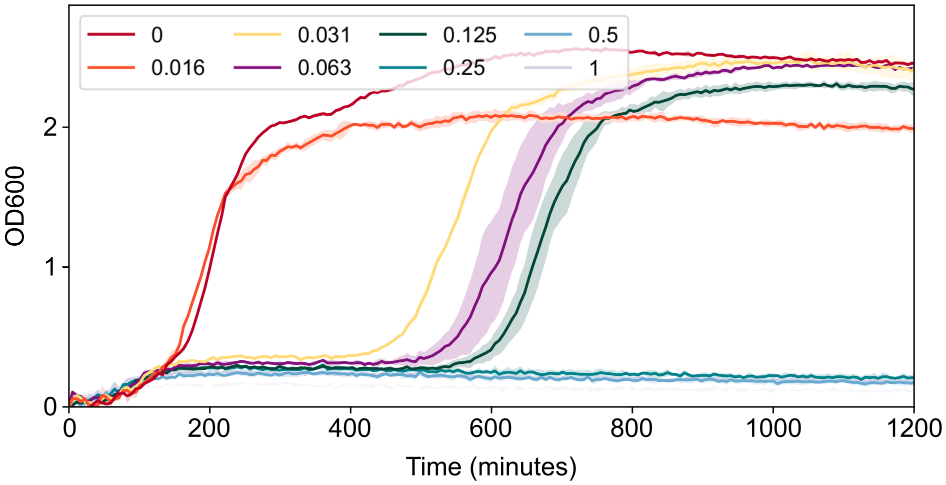

Growth curves for cefotaxime in MOPSgluRDM at 37° C and pH 7. Regrowth is only observed in a narrow cefotaxime concentration range between 0.031 and 0.125μg/ml cefotaxime. Each curve is the average of 2-4 replicates and the shaded areas represent the standard deviation. The blue curve (0.25 μg/ml Ctx.) shows complete inhibition of growth over time, while the orange curve (0.016 μg/ml Ctx.) shows no inhibition.
